# Editorial: Reinforcement learning for real-world robot navigation

**DOI:** 10.3389/frobt.2026.1861947

**Published:** 2026-05-12

**Authors:** Pengqin Wang, Xiaocong Li, Meixin Zhu, Jun Ma

**Affiliations:** 1 Division of Emerging Interdisciplinary Areas, The Hong Kong University of Science and Technology, Hong Kong SAR, China; 2 Agency for Science, Technology and Research (A*STAR), Singapore, Singapore; 3 School of Transportation, Southeast University, Nanjing, China; 4 Robotics and Autonomous Systems Thrust, The Hong Kong University of Science and Technology (Guangzhou), Guangzhou, China

**Keywords:** deep learning, machine learning, motion planning, reinforcement learning, robot navigation

## 1 Introduction

Navigation remains a fundamental challenge for robots across diverse domains, including mobile robots, unmanned aerial vehicles, and autonomous driving systems. Reinforcement learning (RL) has emerged as a powerful paradigm to address these challenges, enabling robots to learn optimal decision-making and motion planning. Benefiting from the trial-and-error interactions with the environments, RL provides unique advantages for dynamic and adaptive navigation. Foundation models, such as large language models and vision transformers, have demonstrated the generalizable capacities across multiple robot tasks. Moreover, their ability to handle multi-modal data and adapt to diverse contexts opens new possibilities for enhancing robot systems. In this case, combining reinforcement learning with foundation models presents exciting opportunities to create more robust, adaptable, and scalable systems, especially in environments that require high-level reasoning or engage in complex decision-making. This Research Topic aims to provide a comprehensive overview of the current state-of-the-art in RL and foundation models for robot navigation, highlighting the latest research, methodologies, and practical implementations.

## 2 Articles of this research topic

Path planning is essential for deploying autonomous robots in complex real-world environments. However, long-term decision-making and sparse rewards propose significant challenges for traditional RL algorithms. Offline hierarchical reinforcement learning (HRL) addresses these issues by decomposing tasks into high-level goal generation and low-level goal navigation. Advanced methods like Guider and HIQL introduce latent spaces to represent subgoals, but their high-level policies must search over continuous latent spaces. Furthermore, these methods can lead to unstable training, especially with policy gradient algorithms like PPO. To overcome this limitation, Han proposes LG-H-PPO, a novel offline hierarchical PPO framework that discretizes the continuous latent space into a structured latent graph. This transforms high-level planning from difficult continuous creation into simple discrete selection, substantially reducing learning complexity. Experiments on D4RL benchmarks show that LG-H-PPO outperforms strong baselines in convergence speed and success rate. The key contribution is integrating graph structures into latent variable HRL planning, which simplifies the high-level action space and improves training efficiency and stability for long-sequence navigation tasks.

Developing reliable DRL navigation policies for mobile robots in highly dynamic real-world environments remains challenging, especially when robots must explore unknown spaces without any prior knowledge while avoiding collisions. Bockrath et al. introduces Trust-Nav, a trustworthy navigation framework that uses variational policy learning to quantify uncertainty in action estimation, localization, and map representation. By applying Bayesian variational approximation to the policy network parameters and combining policy-based with value-based learning, Trust-Nav propagates variational moments through all network layers. Uncertainty in robot actions is measured via propagated variational covariance, while uncertainty in localization and mapping is embedded into the reward function using optimal experimental design theory. Experiments in the Gazebo simulator demonstrate that Trust-Nav achieves robust autonomous navigation and mapping, consistently outperforming deterministic DRL approaches, particularly under noisy conditions and adversarial attacks. By integrating uncertainty into the policy network, Trust-Nav promotes safer, more reliable navigation and represents a step toward self-aware robotic systems that can recognize and respond to their own limitations.

In multi-skill imitation learning for robots, complete expert datasets with full motion features are essential but often unavailable. Datasets based solely on joint positions are more accessible. However, they lack the details for effective skill learning and smooth transitions. To address this, Tu et al. proposes Seamless Multi-Skill Learning (SMSL) framework. Built upon the Adversarial Motion Priors framework and enhanced with self-trajectory augmentation, SMSL leverages high-quality historical experiences to guide skill acquisition and generate natural transitions, overcoming the limitations of incomplete data. An adaptive command sampling mechanism balances training across skills of varying difficulty and prevents catastrophic forgetting. Experiments show that SMSL outperforms baseline methods, and sim-to-real validation on Solo8 robots confirms its effectiveness. This work demonstrates SMSL’s potential for autonomous skill learning from minimal data in real-world robotic applications.

Traditional wilderness search and rescue methods are slow and limited in coverage. The unmanned aerial vehicles offer speed and flexibility, but effective operation requires optimized search paths. Ewers et al. presents a DRL algorithm that generates efficient drone search paths using a probability distribution map of the search area and missing person. The learned policy maximizes the probability of rapid detection. Experimental results show that the proposed method reduces search times by over 160% compared to traditional coverage and search planning algorithms. Furthermore, unlike prior work, this approach employs a continuous action space enabled by cubature, allowing more adaptive flight patterns.

Soft robots offer compliance and human-safe interaction in unstructured environments. However, their soft structures make control algorithm development challenging. Oikonomou et al. introduces a novel motion control technique for a modular bio-inspired soft-robotic arm, focusing on qualitative reproduction of periodic trajectories. The method combines Probabilistic Movement Primitives (ProMP) and Central Pattern Generators (CPG). ProMP provides immediate actuation estimation using a learned library of simple movements without time-consuming training, while CPG filters and parameterizes the resulting signals to generate rhythmic patterns at the motor level. Evaluated on a two-module soft arm, the approach enables rapid acquisition of periodic movements and compression into low-dimensional CPG parameters for long-term storage and execution.

## 3 Conclusion

In summary, the works received highlight significant advances in applying RL and foundation models to robot navigation. Key themes include the integration of hierarchical learning to handle long-term sparse rewards, the use of uncertainty quantification for trustworthy decision-making, the combination of movement primitives with rhythmic pattern generators for soft robot control, and the fusion of DRL with prior knowledge for drone search. In conclusion, the RL approaches provide the optimality of decision-making and the diversity of exploration, while foundation models offer powerful tools for generalization and multi-modal reasoning.

There are several areas of concern. First, bridging the gap between simulation and real-world deployment remains critical. Second, embedding uncertainty awareness into perception and action modules will be essential for safe operation in unpredictable environments. Finally, leveraging foundation models as prior knowledge for low-data adaptation and cross-task transfer presents a potential direction. These approaches will lead to more autonomous, robust, and deployable robotic systems, addressing various challenges in the complex real world.

